# The Mental Health Challenges of Medical Postgraduates in Chengalpattu District, Tamil Nadu: Are they being Educated or Exploited?

**DOI:** 10.7759/cureus.77157

**Published:** 2025-01-08

**Authors:** Anantha Eashwar VM, Arunkumar M, Ilamilaval Thozhanenjan, Ashni Bhandari, Aishwarya PM, Krishna Prasanth, Lakshmi Nagalingam, Nithya V

**Affiliations:** 1 Community Medicine, Sree Balaji Medical College and Hospital, Chennai, IND; 2 Community Medicine, Panimalar Medical College Hospital and Research Institute, Chennai, IND

**Keywords:** comorbid anxiety, faculty development in medical education, healthcare empathy, suicide and depression, time for change, undergraduate and graduate medical education, academic stress

## Abstract

Introduction

Globally, psychological morbidity among medical postgraduates (PGs) is an often overlooked and underreported condition. The study was done to find the prevalence of depression and stress among PGs and to find and explore the associated factors contributing to their stress and depression.

Methodology

The study followed a convergent parallel mixed-method approach, in which qualitative and quantitative data were collected simultaneously. The quantitative component was a cross-sectional study among 224 medical PGs pursuing their course in the Chengalpattu district of Tamil Nadu, India. Depression was assessed using the Patient Health Questionnaire-9 (PHQ-9), and stress was evaluated using the perceived stress scale (PSS). A convenience sampling technique was followed. A pre-tested structured questionnaire was used to collect data regarding their interpersonal relationships with peers, seniors, and faculty members; their sleep patterns; and details regarding their family. Data was collected via an online platform. The qualitative component was collected by three focus group discussions (FGDs) conducted on virtual video calls with eight members in each FGD. The topic guide included semi-structured questions regarding the challenges in their postgraduate course and areas they felt needed improvement in the medical education system. Quantitative data was analyzed using SPSS Statistics version 26 (IBM Corp., Armonk, NY, USA), and qualitative data was collected using QualCoder version 3.5 (Colin C, QualCoder, Massachusetts Institute of Technology, Cambridge, MA, USA) and analyzed using thematic analysis.

Results

The mean age of the study participants was 27± 3 years. The prevalence of depression was found to be 46.4%, and stress was found to be 53.2%. A logistic regression model using the enter method and a linear regression model at a 95% confidence interval were used to find the predictors of depression and stress, respectively, among the PGs. The major predictors of depression were age of less than 27 years, clinical subject stream, less than six hours of sleep per day, inadequate knowledge of the course, and unapproachable faculty in the department. The major predictors of stress included all of the above, along with the feeling of discrimination on the grounds of language and religion. By qualitative analysis, the major themes identified were emotional and mental challenges PGs face in the course, gaps in postgraduate training, exploitation and unfair practices in the department, systematic barriers to education, and recommendations for improvement as perceived by the PGs.

Conclusion

The study found that around half of the study participants were screened positive for depression and stress. Some of the PGs responded that they experienced emotional abuse from seniors and faculties, expressed their need for an unbiased and neutral psychologist on campus, and expressed the need for a proper grievance redressal cell in the institution. At the institutional and departmental levels, measures could be taken to understand the mental health challenges faced by the PGs in an empathetic, non-judgmental, and holistic manner to reduce the psychological morbidity among them.

## Introduction

Postgraduate (PG) medical education is a challenging phase for physicians, as they have to manage their personal lives and economic demands and tend to the academic requirements stipulated by the institution [1]. Managing both personal life and meeting academic demands can lead to stress, which could lead to burnout, depression, anxiety, fatigue, poor sleep, and even substance abuse [2]. There is widespread evidence that physicians who work at reduced physical and intellectual capacity could become harmful to themselves, their colleagues, and their patients [2]. Studies conducted in Western countries on PG medical residents found that around 3% to 35% of them suffer from depression/anxiety during their training period, which could even lead to the development of suicidal ideation [3-6]. However, some studies report an even higher prevalence of depression [7].

The National Medical Commission (NMC) recently issued a report prepared by the National Task Force on the Mental Health and Wellbeing of Medical Students. Among the 5337 PGs surveyed, 31.23% reported having suicidal thoughts, and 4.4% attempted suicide in the last year [8]. Chahal et al., in their study done in India, found from retrospective data obtained from search records using keywords that among the 358 suicide deaths in the medical community between 2010 and 2019, 105 were residents, and the predominant cause was stress [9]. Nearly 20% of PG suicides were caused by harassment in the workplace [10]. The major predictors of mental health among PGs are a history of facing abuse, academic stress, burnout, criticism from seniors and faculties, and family-related stress. The major problems reported by PGs were long working hours, financial instability due to stipends, senior PG students bullying junior PGs, lack of faculty support regarding leave requests, and lack of empathy among faculty [8]. When the mental health of PG physicians is affected, it could impact their work environment and affect the quality of services they provide [11]. Also, it could affect their academic career and learning capacity, leading to increased stress and depression [12]. It has also been found that one-third to one-fourth of medical PGs develop clinical depression at some point in their course of study.

We also need to understand that a certain stress level is required in medical training for effective learning. Stress that could facilitate learning is called favorable stress, and one that can suppress and affect learning is called unfavorable stress. The stressors may also subjectively differ from one PG to another based on their cultural background, coping skills, and background traits [13]. The faculty must understand the nature of the stressors faced by PG students to understand them and prevent untoward consequences regarding their mental health and well-being.

Very few studies have been done to understand the mental health challenges faced by PG medical residents, especially in South India, with none of them exploring the mental health challenges faced by them using qualitative methods. Based on the above background, the primary objectives of the study are (1) to determine the prevalence of depression, stress, and associated factors among PG medical students in Chengalpattu district, Tamil Nadu, India, and (2) to explore the mental health challenges faced by the PGs during their training period. 

## Materials and methods

Study design

The study followed a convergent parallel mixed-method approach, in which qualitative and quantitative data were collected simultaneously and analyzed separately. The quantitative component's study design was cross-sectional. The study was conducted in the Chengalpattu district of Tamil Nadu, India, between October and December 2024. The Institutional Human Ethics Committee of Sree Balaji Medical College and Hospital (Chennai, TN, IND) granted ethical approval (approval no: 002/SBMCH/IHEC/2024/2316).

Quantitative component

Study Population and Sample Size Calculation

The participants were medical PGs studying in various medical colleges across Chengalpattu district. A study by Jain et al. in Maharashtra found that 49.4% of PGs were suffering from depression (mild to severe) [14]. Taking this as prevalence (P) and applying the formula Zα2PQ/L2 where α = 1.96 at a 95% confidence interval, Q = 50.6% (100-P) with an absolute precision (L) of 7%, the minimum required sample size was 204. Accounting for 10% of the non-response rate, the minimum required sample size was 224.

Data Collection

A survey questionnaire was prepared in English and circulated as an online form among social media groups of PGs studying in all medical colleges across the Chengalpattu district. Those who gave consent on the first page of the questionnaire, along with the requested details, were included as study participants. The questionnaire contained questions regarding the sociodemographic details and various questions related to the mental health of PGs. The questionnaire was validated, and a Cronbach's alpha of 0.82 was obtained. Depression was assessed by using the Patient Health Questionnaire-9 (PHQ-9), a validated tool to screen for depression. A score of 5 or above was categorized as having depression [15]. Stress was assessed by using the Perceived Stress Scale (PSS), a validated 10-item questionnaire to measure stress by evaluating the degree to which an individual has perceived their life to be uncontrollable, unpredictable, and overloaded. A score equal to or above 14 was considered as having stress [16].

Regarding online data collection, incomplete responses were deleted, and the data sheet was cleaned before achieving the sample size. No information regarding personal identity, e-mail ID, name, or medical college details was collected at any step of the survey. The online form was closed after receiving a total of 224 responses, which was completed in 2 weeks. The overall response rate was around 22%. 

Data Analysis

Data was analyzed using SPSS Statistics version 26 (IBM Corp., Armonk, NY, USA). The dependent variables were depression, measured on a qualitative scale, and stress, measured on a quantitative scale. The chi-square test was used to check for the association between depression and related variables (sociodemographic variables, variables related to interpersonal relationships with faculty, seniors, and family details). Since stress scores were not normally distributed (according to the Kolmogorov-Smirnov (KS) and Shapiro-Wilk tests), median and interquartile range (IQR) were used to express them descriptively, and the Mann-Whitney U test was used to check for an association between stress scores and related variables. Variables found to be significant in bivariate analysis (chi-square test and independent sample T-test) at a 95% confidence interval were included for logistic regression and a linear regression model for depression and stress, respectively.

Qualitative component

The qualitative data was collected through the virtual platform Zoom (Zoom Communications Inc., San Jose, CA, USA). A virtual call invite was circulated among the social media groups of the PG medical students in the Chengalpattu district with a scheduled date and time. A prescreening questionnaire was used as part of the virtual call invite. It contained the consent to participate, their gender, the branch of medical postgraduation, and instructions on remaining anonymous during the call. During the call, the camera was disabled, and participants were encouraged not to use their profile pictures to remain anonymous. The topic guide was prepared based on the difficulties faced by the PGs during the course and improvements they could suggest for improving PG medical education. It was adapted from the NMC task force report on the mental health and well-being of students [8]. The FGD was started when a group of eight was reached. Similarly, three FGDs were conducted with the primary author and a facilitator, who ensured all participants had equal opportunities to express their views. Data saturation was reached after three FGDs, after which no new themes, subthemes, or codes were obtained. 

All the discussions were recorded and transcribed into text files. The focus groups reconfirmed the trustworthiness of the findings by determining whether their points were interpreted correctly. The transcribed text files were imported into QualCoder version 3.5 (Colin C, QualCoder, Massachusetts Institute of Technology, Cambridge, MA, USA), and each FGD was added under "cases" in the software. The text was coded in the "code text" option, and various themes and subthemes were color-coded to understand and obtain a thematic framework.

## Results

Table 1 shows the demographic and social factors related to the PG course among the study participants. Around 116 (51.8%) of the study participants were above 27 years old and belonged to the clinical stream; 48 (21%) had children. Inadequate sleep was reported in 120 (53%) of the study participants. Regarding discrimination in the department, 28 (12.5%) felt discriminated against based on their native language, and 16 (7.1%) were based on religion and caste/community. Only 144 (64.3%) of the study participants felt they received adequate knowledge in their PG course. Around 120 (53.6%) subjectively stated that they were forced to do personal favors for their superiors in the department. It was observed that 112 (50%) felt emotionally abused by faculty and 64 (28.6%) by seniors.

**Table 1:** Demographic and social factors related to the PG course among the study's participants PG: Postgraduate

No.	Variable	Frequency (n = 224)	Percentage
1	Age		
	< 27 years	108	48.2
	> 27 years	116	51.8
2	PG subject stream		
	Clinical	116	51.8
	Non-clinical	108	48.2
3	Marital status		
	Married	104	46.4
	Unmarried	120	53.6
4	Having children		
	Yes	48	21.4
	No	176	78.6
5	The college gives a stipend.		
	< Rs. 25000	100	44.6
	> Rs.25000	124	55.4
6	Substance use (alcohol/tobacco)		
	Yes	48	21.4
	No	176	78.6
7	Hours of sleep		
	< 6 hours per day	120	53.6
	> 6 hours per day	104	46.4
8	Feel discriminated against based on your native language		
	Yes	28	12.5
	No	196	87.5
9	Feel discriminated against based on religion		
	Yes	16	7.1
	No	208	92.9
10	Feel discriminated against based on caste/community		
	Yes	16	7.1
	No	208	92.9
11	I have adequate knowledge of the PG course in my department.		
	Yes	144	64.3
	No	80	35.7
12	I feel forced/compelled to do personal favors for superiors in my department.		
	Yes	120	53.6
	No	104	46.4
13	Debt in family		
	Yes	116	51.8
	No	108	48.2
14	Cordial relationship with seniors		
	Yes	68	30.4
	No	156	69.6
15	Cordial relationship with co-PGs		
	Yes	40	17.9
	No	184	82.1
16	Felt emotionally abused by seniors		
	Yes	64	28.6
	No	160	71.4
17	Felt emotionally abused by faculty		
	Yes	112	50
	No	112	50
18	The faculty members in the department are approachable in case of any need.		
	Yes	72	32.1
	No	152	67.9
19	At some point, I decided to discontinue the course.		
	Yes	136	60.7
	No	88	39.3

The prevalence of depression in the present study was found to be 46.4%, and stress was found to be 53.2%. Table 2 shows the association between depression and related variables. It was found that an age of less than 27 years, clinical subject stream, discrimination based on caste/community, inadequate sleep, not receiving adequate knowledge during the PG course, and unapproachable faculty were statistically significant predictors of depression on logistic regression analysis with an R2 value of 0.725. Variables like gender were not found to have a statistically significant association with depression and hence were not included in the below table. 

**Table 2:** Logistic regression analysis between depression and related variables *Statistically significant at 95% confidence interval

No.	Variable	Depression		Total (n = 224); n (%)	Unadjusted odd’s ratio (95% CI)	Adjusted odd’s ratio (95% CI)		p-value
		Yes n = 104 n (%)	No n = 120 n (%)					
1	Age							
	<27 years	68 (58.6)	48 (41.4)	116 (51.8)	2.83 (1.64-4.88)	2.70 (1.35-4.15)		0.033*
	>27 years	36 (33.3)	72 (66.7)	108 (48.2)	Reference category			
2	PG subject stream							
	Clinical	72 (62.1)	44 (37.9)	116 (51.8)	3.86 (2.22-6.78)	14.01 (7.22-22.12)		0.000*
	Non-clinical	32 (29.6)	76 (70.4)	108 (48.2)	Reference category			
3	Hours of sleep per day							
	< 6 hours per day	72 (60)	48 (40)	120 (53.6)	3.33 (1.94-5.87)	14.33 (4.88-27.22)		0.000*
	> 6 hours per day	32 (30.8)	72 (69.2)	104 (46.4)	Reference category			
4	Reason for selecting this course							
	Parents compulsion	60 (57.7)	44 (42.3)	104 (46.4)	2.35 (1.37-4.03)	0.62 (0.25-1.53)		0.305
	Out of free will	44 (36.7)	76 (63.3)	120 (53.6)	Reference category			
5	Feel discriminated against based on caste/community							
	Yes	12 (75)	4 (25)	16 (7.1)	3.78 (1.18-12.11)	38.9 (4.82-73.35)		0.000*
	No	92 (44.4)	116 (55.8)	208 (92.9)	Reference category			
6	I have inadequate knowledge of the PG course in my department.							
	Yes	76 (52.8)	68 (47.2)	144 (64.3)	2.07 (1.18-3.64)	1.58 (1.11-2.25)		0.04*
	No	28 (35)	52 (65)	80 (35.7)	Reference category			
7	Cordial relationship with juniors/seniors							
	No	44 (64.7)	24 (35.3)	68 (30.4)	2.93 (1.62-5.30)	1.70 (0.53-5.52)		0.373
	Yes	60 (38.5)	96 (61.5)	156 (69.6)	Reference category			
8	Feeling emotionally abused by seniors							
	Yes	44 (68.8)	20 (31.3)	64 (28.6)	3.66 (1.97-6.80)	0.62 (0.21-1.85)		0.401
	No	60 (37.5)	100 (62.5)	160 (71.4)	Reference category			
9	Feeling emotionally abused by faculty in the department							
	Yes	68 (60.7)	44 (39.3)	112 (50)	3.26 (1.88-5.64)	0.29 (0.09-0.90)		0.32
	No	36 (32.1)	76 (67.9)	112 (50)	Reference category			
10	The faculty members in the department are approachable in case of any need.							
	No	44 (61.1)	28 (38.9)	72 (32.1)	2.41 (1.35-4.38)		10.53 (2.88-20.22)	0.000*
	Yes	60 (39.5)	92 (60.5)	152 (67.9)				
11	At some point, I decided to discontinue the course.							
	Yes	92 (67.6)	44 (32.4)	136 (60.7)	13.23 (6.53-26.85)		4.23 (2.34-7.21)	0.000*
	No	12 (13.6)	76 (86.4)	88 (39.3)				

Table 3 shows the association between stress and related variables. On linear regression analysis, it was found that the major predictors of stress were age less than 27, inadequate sleep, feeling discriminated against based on religion/language, not receiving adequate knowledge during the PG course, and unapproachable faculty. Variables such as gender were not found to have a statistically significant association with stress scores and hence not included in this table. 

**Table 3:** Linear regression analysis between PSS scores and related variables PSS: Perceived Stress Scale *Statistically significant at 95% confidence interval

No.	Variable	PSS score median (IQR)	Mann Whitney U test: p-value	β Co-efficient (95% CI)	p-value
1	Age				
	< 27 years	23 (7)	0.039*	1.652 (0.02 to 3.32)	0.048*
	> 27 years	19 (9)			
2	The college gives a stipend.				
	< Rs. 25,000	24 (9)	0.000*	1.043 (-0.69 to 2.78)	0.238
	> Rs. 25,000	19 (8)			
3	Hours of sleep per day				
	< 6 hours per day	23.5 (9)	0.000*	3.99 (2.21 to 5.76)	0.000*
	> 6 hours per day	18.5 (9)			
4	Reason for selecting this course				
	Parents compulsion	23.5 (9)	0.000*	0.111 (-1.72 to 1.94)	0.905
	Out of free will	19.5 (10)			
5	Feel discriminated against based on your native language				
	Yes	25 (11)	0.048*	3.45 (0.62 to 6.28)	0.017*
	No	20 (9)			
6	Feel discriminated against based on religion				
	Yes	27.5 (17)	0.027*	4.33 (0.55 to 8.11)	0.025*
	No	20 (9)			
7	Feel discriminated against based on caste/community				
	Yes	27 (12)	0.023*	1.47 (-1.81 to 4.77)	0.378
	No	20 (9)			
8	I have inadequate knowledge of the PG course in my department.				
	Yes	24 (11)	0.000*	2.87 (0.81 to 4.94)	0.007*
	No	19 (7)			
9	I feel forced/compelled to do personal favors for superiors in my department.				
	Yes	23 (10)	0.007*	0.253 (-1.55 to 2.06)	0.783
	No	19 (9)			
10	Cordial relationship with juniors/seniors				
	No	25 (6)	0.000*	2.27 (-0.21 to 4.76)	0.073
	Yes	19 (8)			
11	Feeling emotionally abused by seniors				
	Yes	26 (13)	0.000*	-0.78 (-3.21 to 1.65)	0.526
	No	20 (19)			
12	Feeling emotionally abused by faculty in the department				
	Yes	23.5 (8)	0.000*	0.240 (-1.82 to 2.30)	0.819
	No	18 (11)			
13	The faculty members in the department are approachable in case of any need.				
	No	24 (5)	0.000*	3.36 (1.28 to 5.43)	0.002*
	Yes	19 (10)			
14	At some point, I decided to discontinue the course.				
	Yes	24 (9)	0.000*	-0.923 (-3.05 to 1.20)	0.393
	No	18 (6)			

Table 4 shows the significant themes, subthemes, and codes from FGDs. The major categories included emotional and mental health challenges, gaps in PG training, exploitation and unfair practices, systematic barriers to education, and recommendations for improvement. Each theme had three subcategories. The subjective experiences, difficulties, and improvements suggested by the PGs are represented below in quotations under each subcategory and put up as codes in Table 4. 

**Table 4:** Themes, subthemes, and codes obtained from the FGDs FGDs: Focus group discussions; PG: Postgraduate

Themes	Sub-themes	Codes
1. Emotional and mental health challenges	Stress and mental health issues	Criticism in front of peers affects mental health
		Small mistakes lead to judgment and exclusion
		Hierarchical pressure and fear of speaking freely
		The mental toll is due to unethical practices like lying to satisfy seniors.
	Psychological support	Need for a neutral psychologist in medical colleges
		Mentor-mentee system for holistic support
2. Gaps in PG training	Orientation and assessment	Early assessment of emotional intelligence and domain interest
		Proper orientation to expectations at the beginning of the course
		Identifying basic skills required for specialties
	Self-learning challenges	Lack of guidance from faculty
		Bridging gaps in subject knowledge and research skills
		Misconception that PGs must learn independently
	Teaching skills development	Separate training for PGs interested in teaching
		Lack of approachability, empathy, and compassion in untrained faculty
3. Exploitation and unfair practices	Clerical and personal work	PGs are being worked as glorified secretaries in the department
		PGs are out running personal errands for superiors
	Economic exploitation	Buying coffee and refreshments for any PG-related activity in the department
		Customs in the department causing a strain on the financial status
	Favoritism and unequal workload	Certain faculty favor specific PGs, which is unfair.
		Work is distributed unequally among PGs
4. Systemic barriers to education	Hierarchy and bureaucracy	Hierarchical norms in the department cause barriers to effective communication
		The expectation of obedience unthinkingly without asking any questions
5. Recommendations for improvement	Proactive measures	Emotional intelligence and early skill assessment before enrolling in specialties
		Tailored training based on the interests of specific individuals
	Innovative teaching methods	Tailored specialty training could be provided in nodal centers identified by regulatory bodies
	Regulatory oversight	Guidelines for preliminary knowledge and skills in specialties
		Involvement of regulatory bodies in skill assessments and workload management
	Evaluation and curriculum	Continuous assessment by regulatory bodies instead of one final exam
		Simulation-based teaching in all department specialties
		Yearly evaluation of skills and competencies by regulatory bodies

Emotional and mental health challenges

Below are the various statements made by PGs about the 'emotional and mental health challenges' they face in the course of their education on campus and their suggestions on how to tackle these issues.

Stress and Mental Health Issues

"Small mistakes I committed during my course were used against me, and I was judged; I did not get enough chances during surgeries and was criticized in front of all the PGs. It took a huge toll on my mental health, which even made me think of discontinuing the course."

"Whenever I approached the superiors, I was shooed, saying I don’t have the basic knowledge. It puts me down, and it affects my mental health."

"The hierarchy in the department is a major barrier. Every word I speak and express myself has to be carefully weighed when speaking to my superiors. A small misplace of words or not following the hierarchy while speaking with or getting permission from faculty could land me in trouble."

"To survive in my PG course, I had to lie a lot to satisfy my seniors or even for the sake of the faculty if instructed by them. Truth is buried. And lies are the new way of normalcy, which is taking a toll on my moral codes."

“PGs must be treated as professional doctors. We are not mere students”.

"Post-graduation should be a platform for learning and strengthening your knowledge and skills about the field/subject rather than a burden in your life."

Psychological support: "There must be a separate psychologist in the medical college who must be a neutral person unattached to any department," said one student. Another stated that "there could also be a mentor-mentee system for the PGs in which the mentor could be someone other than their guide, who provides holistic support to the PG."

Gaps in Postgraduate Training

Orientation and assessment: "Early assessment, such as a pre-placement examination or an evaluation of emotional intelligence, is essential to determine a postgraduate's compatibility with a specific department. This proactive approach can help prevent potential issues and enhance the postgraduate's learning experience."

"As soon as the PGs join a postgraduate course, their needs and interest in particular domains in the course must be assessed, after which the PG could be trained in those domains, which could give them fulfillment at the end of the course."

"With the advent of the District Residency Program (DRP) and newer things included in the competency-based medical education (CBME) curriculum, it is imperative that the PGs are properly oriented in what is expected of them at the end, at the beginning of the course itself."

Self-learning challenges: "I have always been told that PGs must self-learn, which greatly hindered my learning. I have always been instructed to learn on my own," said one student. "Many PGs join the course years after they complete their undergraduate MBBS. They may not have the necessary knowledge regarding the subject or the research. The faculties could bridge the gap," said another. "National Medical Commission (NMC) could bring forth a set of guidelines in each subject on the basic preliminary knowledge or skills a PG must possess when entering a particular specialty. If the PG lacks those skills, they may be guided on ways to obtain/enrich those skills." A student further said that “the academics are nil. No classes are being taken. Only a seminar where we are roasted, and no one tells us the answer."

Teaching skills development: "I feel that PGs interested in teaching should be provided with separate training before joining as a faculty in a medical college." Also, "if a faculty without adequate teaching skills enrolls in a medical college, it could lead to problems like a lack of approachability, empathy, and compassion when dealing with students, which could, in turn, affect both UGs and PGs."

Exploitation and Unfair Practices

Clerical and personal work: "The PGs are being treated as glorified secretaries in the department, especially in the first year," said one student. "I have bought a saree for one of my faculties in my department with my own money. It has been a customary practice going on in my department," revealed another. “We are used for clerical work. From buying stationary to flowers to whatnot. For even bank-related works, we are sent. What are we? Clerks? I don't know why the department has a clerk, and the government is providing them a salary.”

Economic exploitation: "Despite my weak economic condition, I was forced to buy snacks and coffee during my PG presentations, which would amount to almost Rs. 500 to Rs. 800, depending on the number of faculty members present."

Favoritism and unequal workload: "Favoritism is when certain faculty are compatible with certain PGs. They tend to give less work to them and favor them, which is unacceptable," stated one student. "All the work is being given to PGs. If there is a mistake, it's because of the PGs. From taking classes for UGs to everything, only PGs are working. I don’t know why the others are getting a salary. Take classes. Tell me what to read. Allow us to do our projects and attend conferences.” 

Systemic Barriers to Education

Hierarchy and bureaucracy: "Senior faculty were abusing us, saying that PGs are slaves in the department and must obey blindly without hesitation or opposition," revealed a student. “The senior faculty should be more respectful towards their PGs and show humanity and sensitivity.”

Recommendations for Improvement

Proactive measures: "As soon as the PGs join a postgraduate course, their needs and interest in particular domains in the course must be assessed, after which the PG could be trained in those domains, which could give them fulfillment at the end of the course."

Innovative teaching methods: "Teaching through simulation could be provided for the PGs. If facilities are unavailable in the college, the PGs could be sent to other institutes or nodal centers to receive the necessary education, which the specific state medical council could identify."

Regulatory Oversight

"I feel that my institution could have provided guidelines in each postgraduate subject on the basic preliminary knowledge or skills I must possess when I entered my specialty so that I would have known whether I would have been fit for the course,” admitted one student. 

Evaluation and Curriculum

"A continuous assessment model could be adopted rather than relying solely on a final exam. This approach, which includes both practical and theoretical components, can enhance knowledge retention and provide a more comprehensive evaluation of a postgraduate's learning over three years," stated a student. "If there is a yearly evaluation by the NMC in the form of skills attained, with proper earmarking of competencies every year, then the PGs may not be involved in performing clerical work in the department."

## Discussion

The present study found that almost half of the study participants were found to be depressed (46.4%) and stressed (53.2%). Postgraduate medical education brings various challenges, such as academic, clinical, and research-related work, which may be hectic for individuals trying to balance their personal and professional lives. When multiple factors in the workplace, as discussed in this study, come together, the individual's psychological well-being is affected. The current research among PG medical students has yielded interesting results, which are discussed below.

The prevalence of depression in the present study was 46.4%. A survey by Gogoi et al. in Guwahati found the prevalence to be 37% [17]. A similar study by Prakash et al. conducted in Maharashtra found the prevalence to be 52% [18]. A comparatively lower prevalence of 16.4% and 11.4% were found in studies done by Ingle et al. and Sadiq et al., respectively [19,20]. These variations could be attributed to different cultures and different measuring instruments used to quantify depression and variations in the socio-demographic characteristics between the study populations. 

The present study found that depression was more prevalent among clinical PGs (62.1%) when compared with the non-clinical (29.6%) branch, and the association between depression and the PG subject stream was also found to be statistically significant. Similar results were obtained in a study by Shete et al. in Maharashtra and Dave et al. in Gujarat [21,22]. These findings may be attributed to the fact that clinical PGs, in addition to satisfying the academic requirements, had to deal with patients, late-night duty, and ethical commitments, which could have led to them being more depressed.

Almost 54% of the participants slept for less than six hours per day, and 60% of them were found to be depressed. There was a statistically significant association between inadequate sleep and depression. Similar findings were obtained in a study by Joge et al. in Maharashtra [23]. Due to the hectic work schedule, sleep deprivation could have led to fatigue, reduced level of mental concentration, irritability, and conflicts with seniors and faculties, which could have led to depression among these individuals, as evident from the findings of a study done by Baldwin et al. [24].

Depression and stress scores were significantly higher among those who felt that they had inadequate knowledge regarding the PG course. A review by Sheth et al. found that the current state of the PG curriculum lacks important topics such as health economics and behavioral sciences. Also, due to administrative reasons, PGs are forced to do research and publish papers rather than understand the core research, its impact, values, and outcomes [25]. In an editorial article by Ananthakrishnan, various factors like the archaic examination system, inadequate and lack of training in research methodology and biostatistics, and lack of continuous internal assessment by regulatory bodies have been cited as some of the significant factors that PG medical education is lagging in India [26].

Stress scores were significantly higher among participants who didn’t have a cordial relationship with their colleagues and seniors and those who felt emotionally abused by them. A news article by Nirvana titled 'Untold Story of Postgraduate Medical Education in India' refers to emotional abuse faced by PGs as unspoken but well-known within the medical fraternity. The article reported that seniors would casually engage in non-professional behavior, insulting the junior PGs in front of patients, name-calling and passing unprofessional comments, and ultimately bullying them in the pretext of teaching them [27].

In the qualitative analysis, the major factors, as stated under the theme of emotional and mental health challenges, were problems with the seniors, the department's hierarchy, and unapproachable superiors in case of academic needs. In a study done by Mohan et al., when enquired about their mental health status during their postgraduation, around 18% disagreed they were in a satisfied mental state, and 6.3% strongly disagreed and reported they were under extreme stress and had suicidal thoughts [28]. Regarding mentoring, it was found that around half of the PGs were not taught by faculty during their PG training. Also, only half of the students could interact with the faculty during their training period [28]. It is imperative to understand that PGs must consider all faculty as their mentors and should be free to approach any faculty regarding their academic and personal needs, irrespective of the hierarchical barriers. There could be a grievance cell for the PGs in which they can anonymously report their problems. This could be looked upon by the Medical Educational Unit of the college, which could conduct workshops/meetings for the faculty members, update them regarding the issues faced by the PGs, and suggest measures to address the same.

One of the PGs reported a pre-placement examination and emotional intelligence evaluation to check the compatibility of the PGs in the respective courses. This may not be practically possible for either the institution or regulatory bodies. Instead, each department could develop an orientation program to expose them to the curriculum and benchmarks they need to achieve. If they face any course curriculum or proceedings issues, the faculty could counsel and address them at the course's beginning to prevent further complications.

Most of the PGs reported that the research skills they had to acquire during their postgraduation period were inadequate in publishing articles and completing dissertations. Dissertations submitted by the PGs serve as an essential criterion for assessment during their PG course. A study by Bhawalkar et al. in Pune found that most dissertations reported inadequate research findings and were incomplete without uniformity [29]. This necessitates the need that PGs be oriented appropriately on research methodology as soon as they enter the course, and there have to be checkpoints every four or five months, in which institutional regulatory bodies must monitor the progress and provide guidance on the research work of the PGs. This will not only empower the research skills of the PGs but also provide quality dissertations and publications from the institution.

Under the theme of exploitation and unfair practices, most PGs reported doing clerical work in the department and even running personal errands for their superiors. In an article in the Hindu newspaper regarding medicos in Tamil Nadu, one of the PG students reported that they had to buy tea and snacks for the whole department from a specific shop daily. Also, in clinical departments such as surgery, caste is a significant factor in getting chances in the surgical theaters. It was even quoted that no PG students raised objections because they feared not getting enough training and losing marks in the internal examination. They said they were not supposed to talk or give their opinions or suggestions. They also reported doing tons of paperwork that is not even part of their work schedule. It was also cited that PGs have no separate theory classes and learn by working [30].

Among the recommendations for improvement, the participants reported the need for regulatory oversight and the inclusion of innovative teaching methods in medical education. In a report compiled by Klynveld Peat Marwick Goerdeler (KPMG) India, simulation-based training programs, e-learning, and virtual training modules were suggested to improve medical education. Also, measures like frequent accreditation of medical colleges using standard quality benchmarks and attainment of educational outcomes must be evaluated by comparing an institution's performance with their accreditation status, student satisfaction, and pass percentage in the exams [31]. 

Few of the PGs reported running personal errands for the faculty, buying refreshments for departmental activities, or engaging in activities that involved spending money. It must be understood that faculty must not encourage PGs to do any favors for them for monetary purposes or personal gains. It will give a wrong notion to some PGs who take advantage of faculty and expect some concession regarding attendance and internal marks. This results in favoritism, which is another one of the subthemes reported in the present study [28].

Stress is a common occurrence in all types of education systems. Life is full of challenges and threats. We all need to equip ourselves with the necessary skills to overcome them [32]. Resilience is an individual's competency despite the stressors they face in life [33]. As part of medical education, the NMC task force report emphasizes implementing resilience training programs for medical students in a structured environment with follow-up at regular intervals [8,34]. Though evidence is limited, especially from systematically large-scale studies, it could be tried in medical colleges and observed for the improvement in the coping skills of the students [35].

One of the biggest challenges faced by the faculty is whether to believe if a PG comes to them and says that they are suffering from mental health problems. According to the NMC task force report, around 50% of the faculty believe that students use stress as an excuse to get away from academic-related work [4]. Faculty must understand that the ability to handle stress differs from person to person and is rooted in how they are brought up, the stressors they faced in childhood, and how they have managed them [36]. So before coming to a judgmental opinion that the problem faced by the PG is small/negligible or labeling it as a lie, the faculty could listen to the PG and their problems with empathy and try to make an informed decision before dismissing their concerns without giving any thought. This could foster understanding and cultivate empathy, and PGs will feel free to share their problems, decreasing psychological morbidity.

A major strength of this study is its mixed-method study with qualitative components on mental health challenges faced by medical PGs in Tamil Nadu, as there haven't been any conducted on this topic before. The major limitation of the study is that a non-probability sampling technique was used for data collection in the quantitative component, as an online proforma was circulated. Since the sample size is small and data was collected online, some of the responses may be biased (response bias). Also, for PGs who have responded negatively, it will be difficult to predict the real-world situation without learning the viewpoint of their faculty, as most of the questions in the quantitative component were subjective with dichotomous 'Yes' or 'No' responses. Large-scale studies done among medical college PGs with qualitative data collection from both PGs and faculty could yield better results. 

## Conclusions

Based on the findings of the study, it could be understood that the emotional turmoil the PGs undergo is still untold, and it is happening in some medical colleges, which could ultimately affect their professional development, mental health, and well-being. Continuous competency-based grading of PGs, equipping faculties with teaching programs tailored for PG teaching, and establishment of psychological assessments at regular intervals by unbiased and neutral psychologists are some of the key strategies for intervention. The establishment of clearly set goals and guidelines at institutional and departmental levels, as set forth by the regulatory bodies, to develop a transparent, empathetic, non-judgmental academic environment that could result in the enhancement of the quality of PG medical education are some of the key interventions that could empower PGs to become successful and lead the future health force of our country in their respective specialties.
